# Construction of a High-Density Genetic Map and Identification of Loci Related to Hollow Stem Trait in Broccoli (*Brassic oleracea* L. *italica*)

**DOI:** 10.3389/fpls.2019.00045

**Published:** 2019-01-29

**Authors:** Huifang Yu, Jiansheng Wang, Zhenqing Zhao, Xiaoguang Sheng, Yusen Shen, Ferdinando Branca, Honghui Gu

**Affiliations:** ^1^Institute of Vegetable, Zhejiang Academy of Agricultural Sciences, Hangzhou, China; ^2^Department of Agriculture, Food and Environment, University of Catania, Catania, Italy

**Keywords:** genetic map, SLAF, hollow stems, broccoli, locus

## Abstract

A high-quality genetic map is important for mapping of compound traits. In this study, a genetic map was constructed based on the reference genome TO1000 after specific locus amplified fragment (SLAF) sequencing in a double-haploid segregation population of broccoli, and loci controlling hollow stem trait were identified in the genetic map. The genetic map contains 4,787 SLAF markers, with a mean marker distance of 0.22 cM and the mean sequencing depths of 91.14-fold in the maternal line, 88.97-fold in the paternal line and 17.11-fold in each DH progeny. A locus controlling the hollow stem trait, QHS.C09-2, which could explain 14.1% of the phenotypic variation, was steadily detected on the linkage group nine in the indicated data of 3 years’ trials and BLUE analysis. The genetic map could lay an important foundation for mapping of compound traits, and mapping of hollow stem trait would be basis to clone the genes related to hollow stems in broccoli.

## Introduction

Broccoli (*Brassica oleracea* var. *italica*) is a significant vegetable crop, whose flower heads are rich in vitamins, minerals and secondary metabolites such as glucoraphanin (Bjorkman et al., 2011; Natella et al., 2016; Sarvan et al., 2017). During 2014–2016, global production quantity and area harvested of broccoli and cauliflower (*B. oleracea* L. var. *botrytis*) were both increasing from ∼24.37 to ∼25.32 million tons, from ∼1.29 to ∼1.34 million hectares, respectively^1^. Most of broccoli commercial cultivars were bred by traditional breeding methods in the past decades. Classic breeding methods have constraints and difficulty to analyze compound traits. Modern breeding technology including molecular marker assisted selection (MAS) has merits in analyzing compound traits. High-density genetic maps are important foundation to analyze complex trait and MAS for breeders.

Broccoli is one varietas of *B. oleracea* (Cheng et al., 2016). There have been some mapping populations and associated linkage maps construction in *B.* *oleracea*, such as cabbage, broccoli, and cauliflower (Walley et al., 2012; Wang W. et al., 2012; Zhao et al., 2016), and genetic loci for some traits like leaf trichome and male sterile in cabbage (Liang et al., 2017; Mei et al., 2017) and purple leaf trait in ornamental kale (Liu X. et al., 2017) were mapped correspondingly. The genomes of *B. oleracea* var. *capitata* line 02–12 and kale-like TO1000 were sequenced and assembled successfully (Liu S. et al., 2014; Parkin et al., 2014), which are very important for *B. oleracea* crops. Their genome sequences have been used as reference to construct genetic map in cauliflower, broccoli and ornamental kale (Zhao et al., 2016; Branham et al., 2017; Liu X. et al., 2017).

Hollow stems often happen in broccoli commercial production. Inside some broccoli stems, pith tissues near cavities would become blown or even black. This kind of broccoli flower head loses attraction to consumers. Therefore the occurrence of hollow stems could cause severe economic losses to producers. A hollow stem was considered as a physiological disorder in the inner pith tissues of broccoli (Boersma et al., 2013). Liu (2009) reported that the trait of hollow stems was controlled by multi-genes. But its related genes or loci are still unknown. In the study, a high-quality genetic map was constructed based on the reference genome TO1000 in a broccoli double-haploid (DH) segregation population and loci controlling hollow stem trait were identified in the genetic map.

## Materials and Methods

### Plant Materials and Genomic DNA Extraction

A segregation population with 127 DH lines was achieved by microspore culture of the hybrid which came from a cross between DH lines DH16-2 and DH28-4. The maternal line DH16-2 is big type, and has a heavy flower head, a hollow stem, few branches, small buds, and self compatibility, while the paternal line DH28-4 is compact-type, and has a light flower head, a solid stem, heavy branches, big buds, and strong self incompatibility. The parents and their DH progenies were planted in the Yangdu experimental greenhouse of Zhejiang Academy of Agricultural Sciences and self-pollinated to collect seeds for reserving materials and further phenotype surveying. The phenotype data were from the same individuals assessed three times. In order to avoid the contamination by the pollens from other lines, hands and tools were sterilized with 70 percent ethanol and flowers would be separated by bagging during self-pollination.

Clean young leaves of all offspring and the parent were collected and their whole genomic DNAs were isolated according to the CTAB method. Electrophoresis on 0.8% agarose gel and a spectrophotometer (NanoDrop, United States) were employed to detect the DNA quality and concentration.

### Phenotype Survey and Data Statistics

**FIGURE 1:**
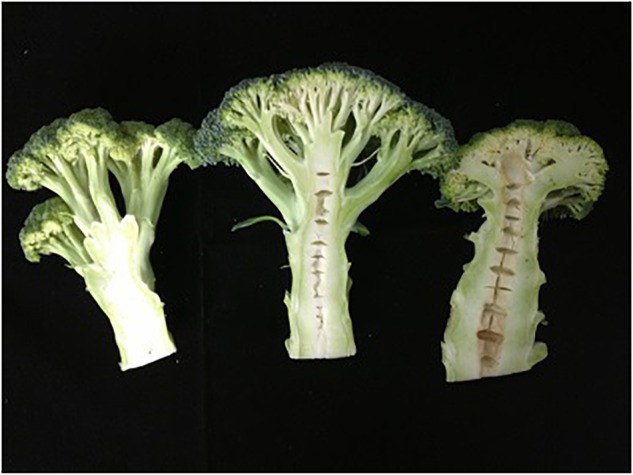
Stem longitudinal sections of maternal line **(Right)**, paternal line **(Left)**, and F_1_ hybrid **(Middle)** of broccoli.

Double-haploid population, the hybrid and their parents were planted in the Yangdu experimental greenhouse in 2014, 2015, and 2017. The overground stem segments were harvested and cut longitudinally in half (Figure 1). Their stem traits were surveyed as hollowness or no-hollowness. There were two replicates per field trial, and there were three plants per replicate. As there were many phenotype data absent due to bad weather in 2014, which affected the QTL analysis using the year’s data. When the environment in different years changes a lot, the phenotype data will have a certain errors or biases and cause that no locus could be steadily detected. Best Linear Unbiased Evaluation (BLUE) is a kind of statistical approach (Liu J. et al., 2017; Pan et al., 2017), which could solve this problem, as it could give the lowest variance of the estimate linear estimators and the errors do not need to be normal, nor do they need to be independent and identically distributed. In this case, the indicated data of 3 years’ trials and BLUE value were employed to analyze QTL. The heritability was calculated as Shen et al. (2018) mentioned.

### SLAF Library Construction and High-Throughput Sequencing

An optimized SLAF-seq strategy was utilized in this study. Two enzymes *Hpy*166II + *Hae* III (New England Biolabs, NEB, United States) were used to digest the genomic DNA from the parent and population individuals. Then, by Klenow Fragment (3′ → 5′ exo-) (NEB) and dATP at 37°C, a single nucleotide (A) overhang was ligated with the digested fragments. Subsequently, the A-tailed fragments were connected to duplex tag-labeled sequencing adapters (PAGE-purified, Life Technologies, United States) by T4 DNA ligase. Polymerase chain reaction (PCR) was carried out using dNTP, diluted digestion-ligation DNA samples, Q5^®^ High-Fidelity DNA Polymerase and PCR primers (Forward primer: 5′- AATGATACGGCGACCACCGA-3′, reverse primer: 5′-CAAGCAGAAGACGGCATACG-3′). Next, PCR products were depurated using Agencourt AMPure XP beads (Beckman Coulter, High Wycombe, United Kingdom) and pooled. Pooled samples were separated by 2% agarose gel electrophoresis. Fragments ranging from 364 to 464 base pairs (with indexes and adaptors) in size were excised and purified using a QIAquick gel extraction kit (Qiagen, Hilden, Germany). Gel-purified products were then diluted. And pair-end sequencing (Each end 125 bp) was performed on an Illumina HiSeq 2500 system (Illumina, Inc., San Diego, CA, United States) at Beijing Biomarker Technologies Corporation.

### SLAF Markers Identification Based on Reference Genome and Genotyping

Specific locus amplified fragment markers were developed and genotyped using procedures described by Sun et al. (2013). Briefly, low-quality reads (quality score < Q30; indicating 0.1% chance of a base error) were filtered out and raw reads left were sorted to each progeny on the basis of duplex barcode sequences. After the barcodes and the terminal 5-bp positions were deleted from each high-quality reads, clean reads from the same sample were mapped onto the *Brassica oleracea* TO1000 genome sequence^2^ using BWA software (Li and Durbin, 2009). Sequences mapped to the same position were defined as one SLAF locus (Zhang et al., 2015). Then, single nucleotide polymorphism (SNP) loci of each SLAF locus were moved out between parents, and SLAFs with more than three SNPs were discarded firstly. Alleles of each SLAF locus were then defined according to parental reads. For diploid species, one SLAF locus can contain at most four genotypes, so SLAF loci with more than four alleles were considered as repetitive SLAFs and then removed. Only SLAFs with two to four alleles were defined as polymorphic and considered as potential markers. All polymorphism SLAFs loci were genotyped with consistency in the parental and offspring SNP loci. The marker codes of the polymorphic SLAFs were analyzed according to the population type DH, which consist of one segregation types (aa × bb).

In order to further ensure the genotyping quality, genotype scoring was executed by a Bayesian approach (Sun et al., 2013). Firstly, *a posteriori* conditional probability was computed using the coverage of each allele and the number of SNP. Then, qualified markers were selected according to genotyping quality score translated from the probability. Low-quality markers for each marker and each individual were counted and the worse markers or individuals were deleted during the dynamic process. When the average genotype quality scores of all SLAF markers reached the cutoff value, the process stopped. High-quality SLAF markers for the genetic mapping were filtered by the following criteria. First, these sequences with depth of the parents below eight folds, or more than five SNPs were deleted. Second, markers which cover genotypes less than 60% of all offspring were discarded. Thirdly, the Chi-squared test was performed to examine the segregation distortion. Markers with significant segregation distortion (*P* > 0.05) were initially excluded from the map construction and were then added later as accessory markers.

### Genetic Map Construction

Markers loci were partitioned primarily into LGs by the modified logarithm of odds (MLOD) scores > 5. Markers with MLOD scores < 5 were wiped out prior to ordering. A newly developed HighMap strategy was used to order the SLAF markers and correct genotyping errors within LGs in order to obtain a high-density and high-quality map (Zhang et al., 2015). Firstly, two-point analysis was used to analyze recombinant frequencies and LOD scores. Then, an iterative process of marker ordering was conducted by simulated annealing algorithms, enhanced Gibbs sampling and spatial sampling (Jansen et al., 2001; Liu D. et al., 2014). At the beginning of the ordering procedure, SLAF markers were chose by spatial sampling. One marker was picked up randomly according to a priority order of test cross, and any marker whose recombination frequency is smaller than a given sampling radius is deleted. Then, simulated annealing was employed to seeking the best map order. Summation of adjacent recombination fractions was obtained referring to Liu S. et al. (2014). When the new map order is rejected in the continuous steps, the annealing system will stop. Multipoint recombination frequencies of the parents were evaluated by blocked Gibbs sampling after the best map order of sample markers were generated (Liu D. et al., 2014). The renewed recombination frequencies were integrated into the map to optimize the map order in the followed cycle of simulated annealing. When a steady map order was obtained, the next map building round would be begun. All the markers were mapped appropriately by the mapping algorithm. Then, the error correction strategy of SMOOTH was disposed according to parental contribution of genotypes (Van Ooijen, 2011), and missing genotypes were imputed by a k-nearest neighbor algorithm (Van Os et al., 2005). Skewed markers were then added into this map by applying a multipoint method of maximum likelihood. Map distances were estimated using the Kosambi mapping function (Huang et al., 2012).

### QTL Analysis

Detection of QTL for hollow stem trait according to the 3 year data was performed by IciMapping v 4.0 software. The phenotype values of the 127 DH lines in 3 years were employed for QTL mapping for multi-environmental trials. The method ICIM-EPI was used for detecting the additive and epistatic QTL. The walking speed was 0.1 cM, and *P* was 0.001 in stepwise regression. The LOD threshold was set by permutation analysis based on 1000 permutations.

BLUE values from 3 years were analyzed by MapQTL 5.0 software. The map was scanned at 1-cM intervals, the maximum LOD with at least greater than two score along the interval was regarded as the location of the QTL, and the area in the LOD score greater than the threshold was regarded as the confidence interval. The LOD score threshold was primitively set at 3.0 for QTL declaration, and a QTL that was greater than this LOD threshold was considered as a potential QTL. If any relevant QTL was recognized, the LOD score threshold was determined using the 1,000-permutation test with a confidence of 0.99. QTLs and LOD scores greater than the threshold at a confidence of 0.99 were declared significant. If no any relevant QTL was identified, the threshold value corresponding to 0.95 of confidence was considered. If still no relevant QTL was identified, 0.90 of confidence was set. If there is no result, the PT test will not be taken into account and the threshold will be lowered to 2.5. If there is no interval for 2.5, the threshold will drop to 2.0.

## Results

### SLAF Sequencing Raw Data Statistics

In this study, leaves from 127 DH individuals in the segregation population and their parent DH lines were employed to extract DNA and further experiments. By counting the proportion of residual restriction sites in reads inserts, the efficiency of normal digestion was 88.54 percent, which indicated that the digestion efficiency was normal. In total, approximate 81.7 G bp data with 408.7 M reads were produced through the high-throughput sequencing, of which 40.03 percent GC (guanine-cytosine) content and 95.02 percent high-quality reads (quality scores > 30) (Table 1). All the raw data has been submitted to National Center of Biotechnology Information (NCBI), the BioProject ID was PRJNA449775.

**Table 1:** Specific locus amplified fragment sequencing raw data of a DH segregation population in broccoli.

Sample ID	Total reads	Total bases	Q30 percentage (%)	GC percentage (%)
Paternal line	14,499,173	2,898,701,480	95.14	39.11
Maternal line	13,467,405	2,692,024,748	94.92	39.10
Offspring	2,998,414	599,248,625	95.02	40.05
Total	408,765,251	81,695,301,688	95.02	40.03


### SLAFs Development According to the Reference Genome

**FIGURE 2:**
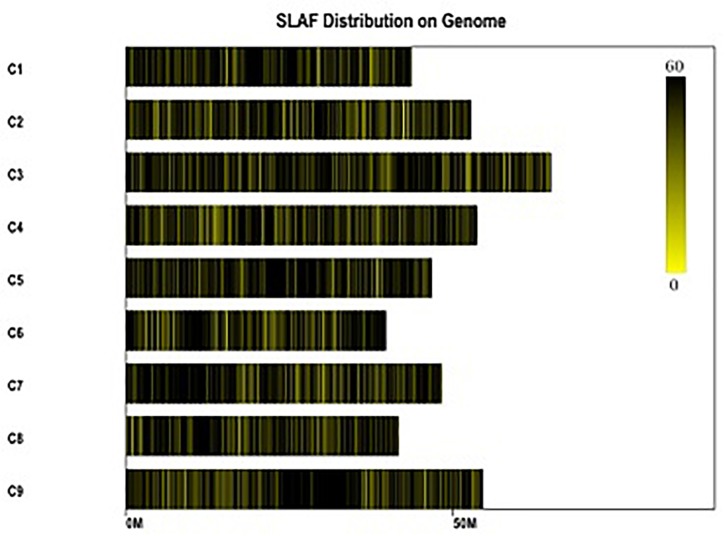
Distribution of the total SLAFs on the reference genome TO1000. The *x*-axis and the *y*-axis represent chromosome length and code, respectively. Each yellow bar stands for a chromosome, and deeper color from yellow to black means more SLAFs on the corresponding location.

**FIGURE 3:**
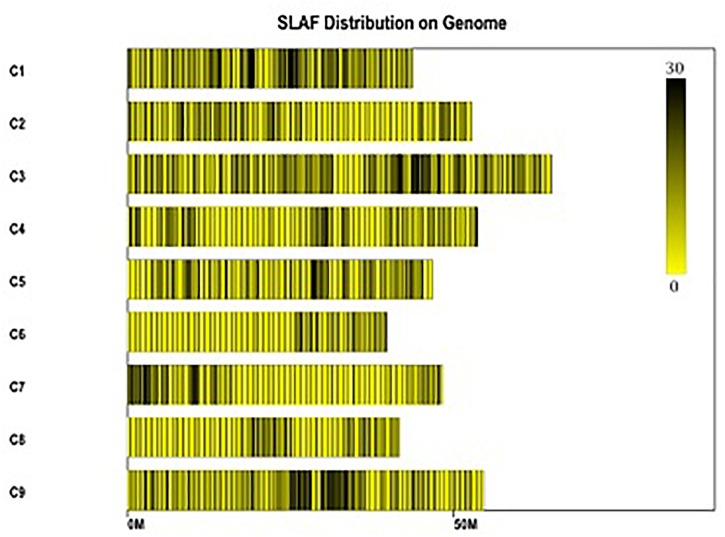
Distribution of polymorphic SLAFs on the reference genome TO1000. The *x*-axis and the *y*-axis represent chromosome length and code, respectively. Each yellow bar stands for a chromosome, and deeper color from yellow to black means more SLAFs on the corresponding location.

All clean reads were aligned with the TO1000 reference genome sequence using BWA software. Among them, 75.99% of all clean reads were mapped on the reference genome. Totally, 24.01% of reads weren’t mapped. 185,349 high-quality SLAFs distributed throughout the reference genome were developed (Figure 2 and Supplementary Table S1). Those SLAFs were located successfully on the reference genome TO1000. The average sequencing depths of these SLAFs were 53.33-, 56.13-, and 12.87-fold in maternal DH line (DH16-2), paternal DH line (DH28-4) and each progeny of the DH population, respectively (Table 2). Then, 185,349 SLAFs were classified as polymorphic, non-polymorphic and repetitive types according to polymorphism analysis of the allele numbers and difference between sequences. 29,215 polymorphic SLAFs were achieved, that’s, 15.76% of the SLAFs are polymorphic (Supplementary Table S2). Polymorphic SLAFs were distributed even on the genome (Figure 3 and Supplementary Table S1). 29,215 polymorphic SLAFs were genotyped with consistency in the parental and DH offspring. Finally, 20,250 polymorphic SLAFs loci were genotyped successfully.

**Table 2:** Sequencing depth of SLAFs in the genetic map.

**High Quality SLAFs**	
No. of SLAFs	185,349
Average depth in maternal line	53.33 ×
Average depth in paternal line	56.13 ×
Average depth in offspring individuals	12.87 ×
**Polymorphic SLAFs OF Maps**	
No. of SLAFs	4787
Average depth in maternal line	91.14 ×
Average depth in paternal line	88.97 ×
Average depth in offspring individuals	17.11 ×


### Map Construction

In order to get a high-quality map, polymorphic SLAFs with lower-quality or more than five SNPs or severe partial separation or covering less 60% separate individuals were filtered. Finally, 4787 high-quality SLAFs were obtained for constructing a genetic map which had the average sequencing depths of 91.14-fold in the maternal line, 88.97-fold in the paternal line and 17.11-fold in each DH progeny (Table 2).

**FIGURE 4:**
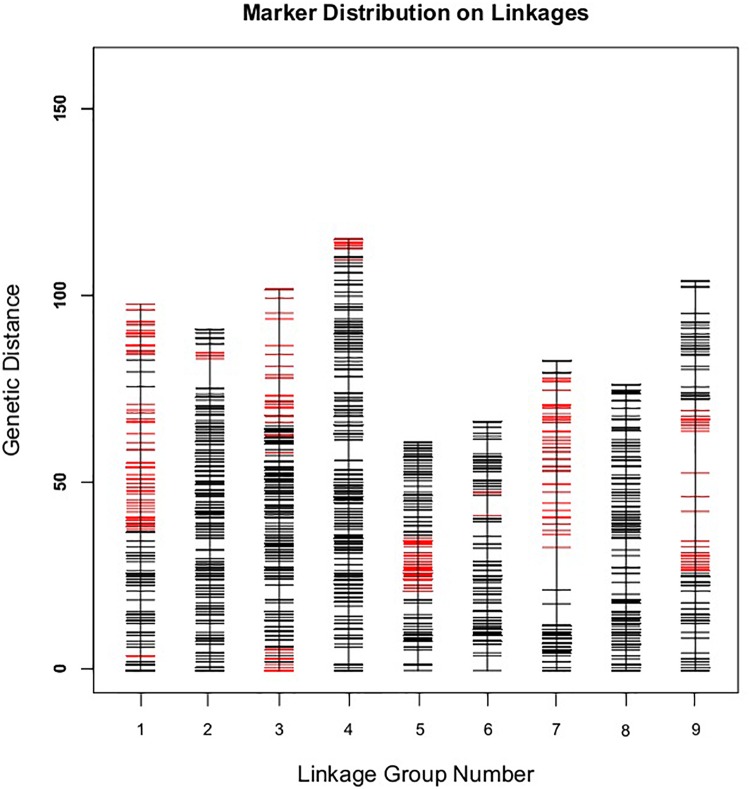
The broccoli genetic map based on the reference genome TO1000. The *x*-and *y*-axis are for linkage group and genetic distance, respectively. Red line in the map is for partial separation markers, and the black one is for normal markers.

**FIGURE 5:**
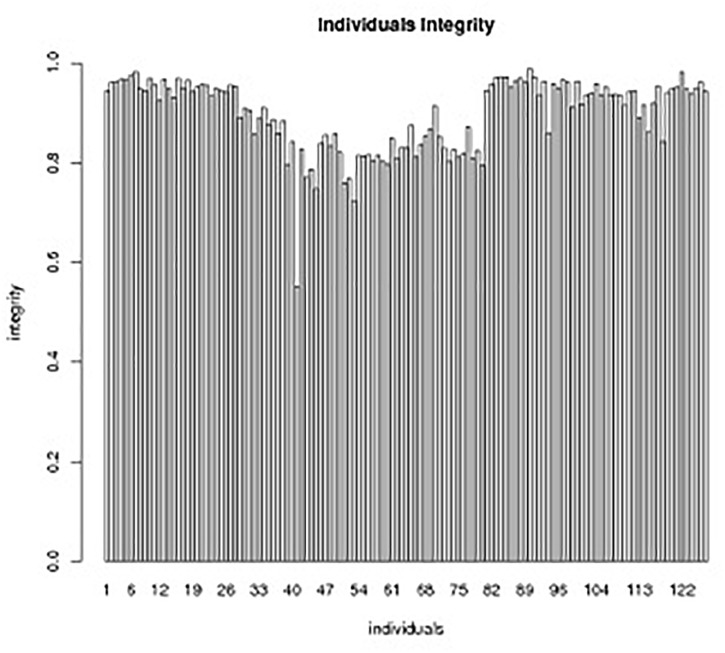
Individual integrity of SLAF markers in the genetic map.The *x*-axis indicates the individual DH lines in the segregation group, and the *y*-axis shows the number of markers.

**FIGURE 6:**
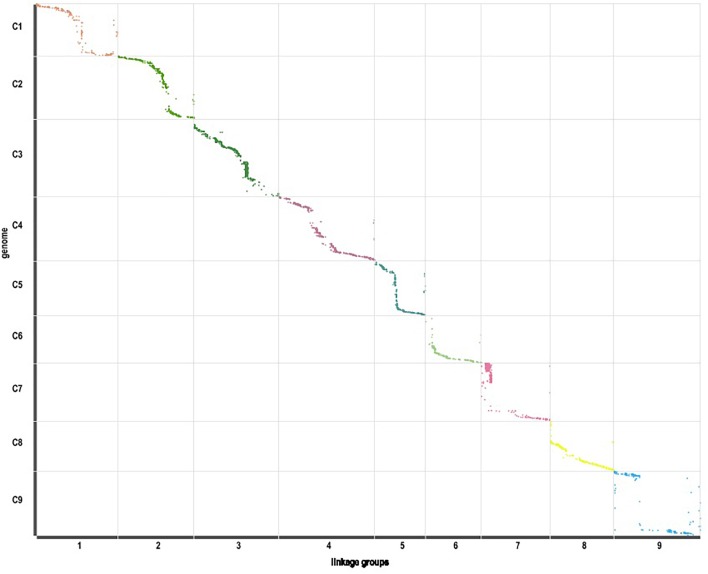
Linearity comparison between the markers in the genetic map and sequences in nine pseudo-chromosomes of TO1000 genome.

Those markers with MLOD values less than three were filtered out. Then, all 4787 SLAFs were distributed even on nine LGs. Linear arrangements of markers among each linkage group and genetic distances between them were analyzed using Highmap software. Eventually, a genetic map covering the reference genome 442.34 Mb with total genetic distance 798.61 cM was constructed (Figure 4 and Table 3). This map contained 9,367 SNPs, with an average genetic distance 0.22 cM and a max gap 16.45 cM. There were gaps larger than 10 cM on LG7 and LG9. And there were 768 markers showing segregation distorting, 0.02 percent of singleton and 1.75 percent of miss. The average markers integrity of the map reached 90.00% (Figure 5). The markers in the genetic map had high colinearity with the reference genome TO1000 (Figure 6).

**Table 3:** Basic information of the genetic map.

LG ID	1	2	3	4	5	6	7	8	9	Total
Total SLAFs	277	675	1096	760	442	344	495	488	210	4787
Total SNPs	550	1442	1990	1519	885	657	1041	899	384	9367
Size (cM)	98.11	91.38	102.03	115.46	61.13	66.67	82.95	76.56	104.32	798.61
Physical length (Mb)	43.49	51.96	64.90	53.69	45.31	39.81	47.92	41.63	53.63	442.34
Average Distance	0.36	0.14	0.09	0.15	0.14	0.19	0.17	0.16	0.54	0.22
Gaps < = 5	1	0.9985	0.9991	0.9987	1	0.9971	0.9937	1	0.9784	0.9962
Max gap	4.74	7.94	7.13	5.53	3.95	5.53	16.45	3.15	13.72	16.45
Total DS	143	5	238	51	221	5	53	0	52	768
Singleton(%)	0	0	0	0	0	0	0.02	0	0	0.02
Miss (%)	0.17	0.24	0.4	0.26	0.04	0.11	0.45	0.08	0	1.75


### QTLs of Hollow Stem Trait in Broccoli

Broccoli stems trait in the DH population was surveyed in 3 years. The stem traits were surveyed as hollowness or no-hollowness in 3 years. The correlation of phenotypic data in the DH group in 3 years was analyzed. The correlation between 2014s data and 2015s data was 0.805, one between 2014s and 2017s was 0.726, one between 2015s and 2017s was 0.817. The heritability of hollow stem trait was about 71% (Supplementary Table S3).

**FIGURE 7:**
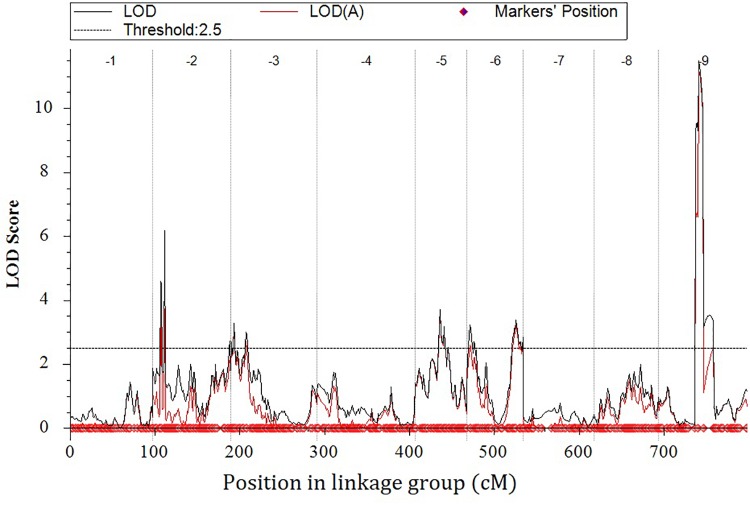
Genome-wide scan using the integrated data.

**FIGURE 8:**
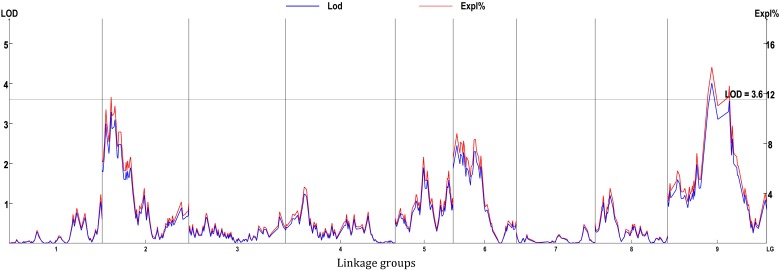
Genome-wide scan using BLUE values.

Eight locations for hollow stems trait were recognized: three loci were on LG2 (QHS.C02-1, QHS.C02-2, and QHS.C02-3), two on LG3 (QHS.C03-1 and QHS.C03-2) and another three loci on LG3, 6, 9 (QHS.C03, QHS.C06, and QHS.C09), respectively (Figure 7). Each locus in the first seven loci could explain less than 10% of the phenotypic variation, while the eighth locus (QHS.C09) could explain 15.6%. Additive effect of each locus among eight loci was very weak (Table 4). And one locus, QHS.C09-2, which could explain 14.1% of the phenotypic variation, was detected in the analysis using BLUE values (Table 4 and Figure 7, 8). This locus was steadily detected in the indicated data of 3 years’ trials and BLUE analysis (Figure 8).

**Table 4:** Quantitative trait loci (QTLs) related with hollow stem trait in a doubled haploid broccoli population (*N =* 127) evaluated in field trials in 2014, 2015, and 2017.

QTL	Range (cM)	Range (Mb)	LOD	Add^a^	%PVE^b^
^c^QHS.C02-1	13.397–14.972	1.482–1.716	6.17	0.085	7.7
^c^QHS.C02-2	8.670–9.458	1.766–2.354	4.58	0.081	6.3
^c^QHS.C02-3	90.413–91.379	51.363–51.920	2.72	0.067	3.2
^c^QHS.C03-1	3.939–4.761	9.260–10.007	3.28	–0.080	4.1
^c^QHS.C03-2	15.826–18.190	13.157–16.000	2.99	–0.072	3.6
^c^QHS.C05	29.939–30.726	40.940–40.993	3.69	–0.085	4.7
^c^QHS.C06	3.946–4.733	23.907–24.509	3.23	–0.070	4.3
^c^QHS.C09-1	46.551–52.883	49.975–50.160	11.48	–0.170	15.6
^d^QHS.C09-2	46.551	49.975	4.01	0.112	14.1


*^*a*^Percent of the phenotypic variation explained by the QTL. ^*b*^Additive effect of scanning position. ^*c*^QTLs associated to hollow stem traits evaluated in 2014, 2015, and 2017. ^*d*^QTLs according to the best linear unbiased estimator (BLUE) values.*

## Discussion

High-quality genetic maps are important for identifying genes of complex traits. The genomes of *B. oleracea* were sequenced and assembled successfully (Liu D. et al., 2014; Parkin et al., 2014). In this study, the markers in the genetic map have high collinearity with the reference genome TO1000, which indicated that it was reliable that SLAFs were developed and the genetic map was constructed based on the reference genome. However, 75.99% of all clean reads were mapped on the reference genome and 24.01% reads couldn’t be mapped on it. Probably, because one genotype in a species sequenced with high-resolution couldn’t contain all sequences information of this species. The reference genome TO1000 could not cover all sequences of *B. oleracea*. For example, even in the two elite indica rice varieties’ genomes ZS97RS1 and MH63RS1, there were 1,891 non-TE genes present in the ZS97RS1 genome but not sequenced in the MH63RS1 genome, and conversely 2,957 non-TE genes present in the MH63RS1 genome but not sequenced in ZS97RS1; and there were a surprisingly large number of structural variations including inversions, translocations, and presence/absence variations between them (Zhang et al., 2016). Broccoli and kale-like line TO1000 are two different varietas in *B. oleracea*. They have remarkable phenotype differences. Among them, the most important difference is that broccoli has a big flower head and TO1000 hasn’t. So, there might be many differences in genome sequences and structural variations between the two varietas, which could cause some reads in broccoli couldn’t be mapped on the reference genome.

The genetic map was constructed by a DH segregate group attained by microspore culture. In microspore culture, production of embryos and regenerated plants depend on genotypes to a certain degree in *B. oleracea* (Gu et al., 2014), which could affect construction of a genetic map. Here, the average marker interval of the map was as short as 0.22 cM, however, there were still two gaps more than 10 cM, which were on the LG7 and LG9, respectively. And there were a certain amount of distorted markers in this genetic map. The reason for them is presumably that microspore culture might select a certain genotypes to produce embryos, which could cause segregation distort and no individuals with recombination event on the two genome intervals in the segregation population.

The heritability of hollow stem trait was about 71%, which showed that although the hollow stem trait was genetic, it was affected by environments. It was consistent with our observation over years and other’s finding (Boersma et al., 2013). During the survey of the six generations (maternal and paternal line, hybrid F_1_, F_2_, and two backcross groups), it was observed that high temperature and drought could make hollowness serious, while low temperature and enough water could reduce the hollowness. But in all environments tested, maternal line DH16-2 was always hollow and paternal line DH28-4 was never hollow. These observations and the result in this study could show that the hollowness in broccoli stems was controlled by genes and meanwhile affected by external environment. Besides, hollow stems also occur in other vegetable crops such as tomato and beans (Aloni and Pressman, 1981; Huberman et al., 1993; Carr and Jaffe, 1995). Drought stress and exogenous plant growth regulation such as ethephon would induce stems hollow (Huberman et al., 1993). Hollow stems were found to develop just after inflorescence initiation in broccoli, then stems enlarged rapidly and starch content in the pith decreased (Boersma et al., 2013). Boron deficiency was blamed for the underlying cause of hollow stem in broccoli and cauliflower crops (Shelp and Shattuck, 1987). However, Boersma et al. (2013) didn’t observe many symptoms associated with a boron deficiency in broccoli and cauliflower. No consistent results and relative genes on hollow stems in broccoli and cauliflower were found.

Hollow stem trait in broccoli is significant in production and was reported 50 years ago (Boersma et al., 2013). But, this trait in stem is complex, and the relevant physiological and genetic research had rarely been reported, potentially because it was difficult to measure the phenotype of the hollow stem. In this study, the phenotype was simply recorded as hollowness and no-hollowness in the 3 years’ evaluation. Accordingly eight loci were identified for the hollow-stem trait. But most of them could explain very low phenotypic variation. Only one locus, QHS.C09-1, could explain higher phenotypic variation, about 15.6 percent. And this locus also partly overlapped with the locus, QHS.C09-2, which was identified using BLUE values. Therefore QHS.C09-2 was presumed to be the major locus related to hollow stem in broccoli.

In this study, though a major locus was detected, there are still many queries which need to be uncovered in the future. For example, cavities in broccoli hollow stems were irregular (Boersma et al., 2013), such as some are short in transection width but long in longitudinal direction, while some the opposite. Is there any possibility that different locus or gene would control, respectively, transection width and longitudinal direction of a cavity. Maybe, the method to measure shape of rice grain, which has been evaluated by grain length (GL), grain width (GW), and grain thickness (GT), could be referred to in future study. Many different QTLs controlling them in rice have been identified and some genes related to GL, GW and GT have been cloned, respectively (Fan et al., 2006; Song et al., 2007; Weng et al., 2008; Li et al., 2011; Wang S.K. et al., 2012; Zhang et al., 2012).

## Author Contributions

HY and HG designed and performed the experiments. JW surveyed the phenotype. ZZ were involved in the data analysis. XS and YS performed the microspore culture. FB was a cooperator in the European project.

## Conflict of Interest Statement

The authors declare that the research was conducted in the absence of any commercial or financial relationships that could be construed as a potential conflict of interest.

## Footnotes

1 http://www.fao.org/faostat/en/#data/QC

2 http://plants.ensembl.org/Brassica_oleracea

## References

[B1] Aloni B. Pressman E. (1981). Stem pithiness in tomato plants: the effect of water stress and the role of abscisic acid. *Physiol. Plant* 51 39–44. 10.1111/j.1399-3054.1981.tb00876.x

[B2] Bjorkman M. Klingen I. Birch A. N. E. Bones A. M. Bruce T. J. A. Johansen T. J. (2011). Phytochemicals of brassicaceae in plant protection and human health-influences of climate, environment and agronomic practice. *Phytochemistry* 72 538–556. 10.1016/j.phytochem.2011.01.014 21315385

[B3] Boersma M. Gracieb A. J. Brown P. H. (2013). Evidence of mechanical tissue strain in the development of hollow stem in broccoli. *Sci. Hortic.* 164 353–358. 10.1016/j.scienta.2013.09.020

[B4] Branham S. E. Stansell Z. J. Couillard D. M. Farnham M. W. (2017). Quantitative trait loci mapping of heat tolerance in broccoli (*Brassica oleracea* var. italica) using genotyping-by-sequencing. *Theor. Appl. Genet.* 130 529–538. 10.1007/s00122-016-2832-x 27900399

[B5] Carr S. M. Jaffe M. J. (1995). Pith autolysis in herbaceous, dicotyledonous plants: experimental manipulation of pith autolysis in several cultivated species. *Ann. Bot.* 75 587–592. 10.1006/anbo.1995.1063

[B6] Cheng F. Sun R. Hou X. Zheng H. Zhang F. Zhang Y. (2016). Subgenome parallel selection is associated with morphotype diversification and convergent crop domestication in *Brassica rapa* and *Brassica oleracea*. *Nat. Genet.* 48 1218–1224. 10.1038/ng.3634 27526322

[B7] Fan C. Xing Y. Mao H. Lu T. Han B. Xu C. (2006). *GS3*, a major QTL for grain length and weight and minor QTL for grain width and thickness in rice, encodes a putative transmembrane protein. *Theor. Appl. Genet.* 112 1164–1171. 10.1007/s00122-006-0218-1 16453132

[B8] Gu H. Zhao Z. Sheng X. Yu H. Wang J. (2014). Efficient doubled haploid production in microspore culture of loose-curd cauliflower (*Brassica oleracea* var. botrytis). *Euphytica* 195 467–475. 10.1007/s10681-013-1008-x

[B9] Huang X. Zhao Y. Wei X. Li C. Wang A. H. Zhao Q. (2012). Genome-wide association study of flowering time and grain yield traits in a worldwide collection of rice germplasm. *Nat. Genet.* 44 32–39. 10.1038/ng.1018 22138690

[B10] Huberman M. Pressman E. Jaffe M. (1993). Pith autolysis in plants: IV. The activity of polygalacturonase and cellulase during drought stress induced pith autolysis. *Plant Cell Physiol.* 34 795–801.

[B11] Jansen J. De Jong A. Van Ooijen J. (2001). Constructing dense genetic linkage maps. *Theor. Appl. Genet.* 102 1113–1122. 10.1007/s001220000489

[B12] Li H. Durbin R. (2009). Fast and accurate short read alignment with burrows-wheeler transform. *Bioinformatics* 25 1754–1760. 10.1093/bioinformatics/btp324 19451168 PMC2705234

[B13] Li Y. B. Fan C. C. Xing Y. Z. Jiang Y. H. Luo L. J. Sun L. (2011). Natural variation in *GS5* plays an important role in regulating grain size and yield in rice. *Nat. Genet.* 43 1266–1269. 10.1038/ng.977 22019783

[B14] Liang J. L. Ma Y. Wu J. Cheng F. Liu B. Wang X. W. (2017). Map-based cloning of the dominant genic male sterile *Ms-cd1* gene in cabbage (*Brassica oleracea*). *Theor. Appl. Genet.* 130 71–79. 10.1007/s00122-016-2792-1 27704179

[B15] Liu E. (2009). *Genetic Dissection and ssr Analysis of Head Appearance Quality Traits in Broccoli (Brassica oleracea var. Italica)*. Beijing: Chinese academy of agricultural sciences.

[B16] Liu J. Huang J. Guo H. Lan L. Wang H. Xu Y. (2017). The conserved and unique genetic architecture of kernel size and weight in maize and rice. *Plant Physiol.* 175 774–785. 10.1104/pp.17.00708 28811335 PMC5619898

[B17] Liu D. Ma C. Hong W. Huang L. Liu M. Liu H. (2014). Construction and analysis of high-density linkage map using high-throughput sequencing data. *PLoS One* 9:e98855. 10.1371/journal.pone.0098855 24905985 PMC4048240

[B18] Liu S. Liu Y. Yang X. Tong C. Edwards D. Parkin I. A. (2014). The *Brassica oleracea* genome reveals the asymmetrical evolution of polyploid genomes. *Nat. Commun.* 5:3930. 10.1038/ncomms4930 24852848 PMC4279128

[B19] Liu X. Gao B. Han F. Fang Z. Yang L. Zhuang M. (2017). Genetics and fine mapping of a purple leaf gene, *BoPr*, in ornamental kale (*Brassica oleracea* L. var. acephala). *BMC Genomics* 18:230. 10.1186/s12864-017-3613-x 28288583 PMC5348804

[B20] Mei J. Wang J. Li Y. Tian S. Wei D. Shao C. (2017). Mapping of genetic locus for leaf trichome in *Brassica oleracea*. *Theor. Appl. Genet.* 130 1953–1959. 10.1007/s00122-017-2936-y 28634808

[B21] Natella F. Maldini M. Nardini M. Azzini E. Foddai M. S. Giusti A. M. (2016). Improvement of the nutraceutical quality of broccoli sprouts by elicitation. *Food Chem.* 201 101–109. 10.1016/j.foodchem.2016.01.063 26868554

[B22] Pan Q. Xu Y. Li K. Peng Y. Zhan W. Li W. (2017). The genetic basis of plant architecture in 10 maize recombinant inbred line populations. *Plant Physiol.* 175 858–873. 10.1104/pp.17.00709 28838954 PMC5619899

[B23] Parkin I. A. Koh C. Tang H. Robinson S. J. Kagale S. Clarke W. E. (2014). Transcriptome and methylome profiling reveals relics of genome dominance in the mesopolyploid *Brassica oleracea*. *Genome Biol.* 15:R77. 10.1186/gb-2014-15-6-r77 24916971 PMC4097860

[B24] Sarvan I. Kramer E. Bouwmeester H. Dekker M. Verkerk R. (2017). Sulforaphane formation and bioaccessibility are more affected by steaming time than meal composition during *in vitro* digestion of broccoli. *Food Chem.* 214 580–586. 10.1016/j.foodchem.2016.07.111 27507513

[B25] Shelp B. J. Shattuck V. I. (1987). Boron nutrition and mobility, and its relation tohollow stem and the elemental composition of greenhouse grown cauliflower. *J. Plant Nutr.* 10 143–162. 10.1080/01904168709363564

[B26] Shen Y. Yang Y. Xu E. Ge X. Xiang Y. Li Z. (2018). Novel and major QTL for branch angle detected by using DH population from an exotic introgression in rapeseed (*Brassica napus* L.). *Theor. Appl. Genet.* 131 67–78. 10.1007/s00122-017-2986-1 28942459

[B27] Song X. J. Huang W. Shi M. Zhu M. Z. Lin H. X. (2007). A QTL for rice grain width and weight encodes a previously unknown ring-type E3 ubiquitin ligase. *Nat. Genet.* 39 623–630. 10.1038/ng2014 17417637

[B28] Sun X. Liu D. Zhang X. Li W. Liu H. Hong W. (2013). SLAF-seq: an efficient method of large-scale *de novo* SNP discovery and genotyping using high-throughput sequencing. *PLoS One* 8:e58700. 10.1371/journal.pone.0058700 23527008 PMC3602454

[B29] Van Ooijen J. (2011). Multipoint maximum likelihood mapping in a full-sib family of an outbreeding species. *Genet. Res.* 93 343–349. 10.1017/S0016672311000279 21878144

[B30] Van Os H. Stam P. Visser R. G. Van Eck H. J. (2005). Smooth: a statistical method for successful removal of genotyping errors from high-density genetic linkage data. *Theor. Appl. Genet.* 112 187–194. 10.1007/s00122-005-0124-y 16258753

[B31] Walley P. G. Carder J. Skipper E. Mathas E. Lynn J. Pink D. (2012). A new broccoli × broccoli immortal mapping population and framework genetic map: tools for breeders and complex trait analysis. *Theor. Appl. Genet.* 124 467–484. 10.1007/s00122-011-1721-6 22038485 PMC3608877

[B32] Wang S. K. Wu K. Yuan Q. B. Liu X. Y. Liu Z. B. Lin X. Y. (2012). Control of grain size, shape and quality by *OsSPL16* in rice. *Nat. Genet.* 44 950–954. 10.1038/ng.2327 22729225

[B33] Wang W. Huang S. Liu Y. Fang Z. Yang L. Hua W. (2012). Construction and analysis of a high-density genetic linkage map in cabbage (*Brassica oleracea* L. var. capitata). *BMC Genomics* 13:523. 10.1186/1471-2164-13-523 23033896 PMC3542169

[B34] Weng J. Gu S. Wan X. Gao H. Guo T. Su N. (2008). Isolation and initial characterization of GW5, a major QTL associated with rice grain width and weight. *Cell Res.* 18 1199–1209. 10.1038/cr.2008.307 19015668

[B35] Zhang J. Chen L. Xing F. Kudrna D. A. Yao W. Copetti D. (2016). Extensive sequence divergence between the reference genomes of two elite indica rice varieties Zhenshan 97 and Minghui 63. *PNAS* 113 E5163–E5171. 10.1073/pnas.1611012113 27535938 PMC5024649

[B36] Zhang J. Zhang Q. Cheng T. Yang W. Pan H. Zhong J. (2015). High-density genetic map construction and identification of a locus controlling weeping trait in an ornamental woody plant (*Prunus mume* Sieb. et Zucc). *DNA Res.* 22 183–191. 10.1093/dnares/dsv003 25776277 PMC4463843

[B37] Zhang X. J. Wang J. F. Huang J. Lan H. X. Wang C. L. Yin C. F. (2012). Rare allele of *OsPPKL1* associated with grain length causes extra-large grain and a significant yield increase in rice. *Proc. Natl. Acad. Sci. U.S.A.* 109 21534–21539. 10.1073/pnas.1219776110 23236132 PMC3535600

[B38] Zhao Z. Gu H. Sheng X. Yu H. Wang J. Huang R. (2016). Genome-wide single-nucleotide polymorphisms discovery and high-density genetic map construction in cauliflower using specific-locus amplified fragment sequencing. *Front. Plant Sci.* 7:334. 10.3389/fpls.2016.00334 27047515 PMC4800193

