# Supplementary material for: Construction of a High-Density Genetic Map and Identification of Loci Related to Hollow Stem Trait in Broccoli (Brassic oleracea L. italica)
Source: Front Plant Sci. 2019 Jan 29;10:45. doi: 10.3389/fpls.2019.00045 (PMC6361793; doi:10.3389/fpls.2019.00045)
Supplement: Supplementary file 1 [file Table_1.docx]

Table S1. Statistics of SLAFs and polymorphic SLAFs Distribution on the linkage groups

| Chr ID | SLAF Number | Polymorphic SLAF |
| --- | --- | --- |
| 1 | 17,744 | 3,759 |
| 2 | 19,473 | 2,753 |
| 3 | 24,929 | 4,931 |
| 4 | 19,687 | 2,685 |
| 5 | 18,865 | 2,854 |
| 6 | 15,042 | 1,585 |
| 7 | 19,140 | 2,357 |
| 8 | 16,209 | 1,839 |
| 9 | 21,638 | 3,966 |
| Other | 12,622 | 2,486 |
| Total | 185,349 | 29,215 |

Note: Chr ID：Code of chromosome; SLAF Number：the number of SLAFs；Polymorphic SLAF：the number of polymorphic SLAFs; Other: the number of SLAFs distributed on scaffolds.
