# Supplementary material for: Construction of a High-Density Genetic Map and Identification of Loci Related to Hollow Stem Trait in Broccoli (Brassic oleracea L. italica)
Source: Front Plant Sci. 2019 Jan 29;10:45. doi: 10.3389/fpls.2019.00045 (PMC6361793; doi:10.3389/fpls.2019.00045)
Supplement: Supplementary file 2 [file Table_2.docx]

Table S2 Types statistics of SLAFs identified based on the reference genome.

| Type | Polymorphic SLAF | Non-Polymorphic SLAF | Repetitive SLAF | Total SLAF |
| --- | --- | --- | --- | --- |
| Number | 29,215 | 155,579 | 555 | 185,349 |
| Percentage | 15.76% | 83.94% | 0.30% | 100% |

Note: Polymorphic SLAF indicates that there is polymorphism site in a SLAF tag , while the polymorphism site mainly includes SNP and InDel; Non-Polymorphic SLAF indicates that there is no polymorphic locus in the SLAF tag; Repetitive SLAF refers to SLAF tags located in the repeating sequence region; Total SLAF is all types of SLAF tags.
