# Supplementary material for: Construction of a High-Density Genetic Map and Identification of Loci Related to Hollow Stem Trait in Broccoli (Brassic oleracea L. italica)
Source: Front Plant Sci. 2019 Jan 29;10:45. doi: 10.3389/fpls.2019.00045 (PMC6361793; doi:10.3389/fpls.2019.00045)
Supplement: Supplementary file 3 [file Table_3.docx]

Table S3 The heritability of hollow stem trait

|  | DF | SS | MS | F value | h^2^ |
| --- | --- | --- | --- | --- | --- |
| Genotype | 115 | 2284.48 | 19.86504 | 4.240319 | 0.709877 |
| Environment | 2 | 12.42 | 6.21 | 1.325564 |  |
| G*E | 230 | 1325.56 | 5.763304 | 1.230214 |  |
| Error | 348 | 1630.31 | 4.684799 |  |  |
